# A Tale of Three Rarities: Secondary Amyloid A (AA) Amyloidosis Caused by Recurrent Sialadenitis and Complicated by Pulmonary Hypertension and Adrenal Insufficiency

**DOI:** 10.7759/cureus.7792

**Published:** 2020-04-23

**Authors:** Parth Desai, Chimezie Mbachi, Udit Joshi, Benjamin Mba

**Affiliations:** 1 Internal Medicine, John H. Stroger, Jr. Hospital of Cook County, Chicago, USA; 2 Cardiology, OSF Healthcare, Peoria, USA

**Keywords:** secondary amyloidosis, hematology, adrenal insufficiency, pulmonary hypertension

## Abstract

A 48-year-old lady presented with a parotid mass found to be secondary to recurrent sialadenitis. She was also found to have microcytic anemia, renal dysfunction, an elevated gamma gap, and an isolated alkaline phosphatase elevation. Later, she developed altered mental status and shock, and was found to have adrenal insufficiency, pulmonary hypertension, and pulmonary nodules. A liver biopsy was consistent with amyloid deposition. The constellation of findings was consistent with systemic amyloid A (AA) amyloidosis secondary to recurrent sialadenitis with hepatic, renal, pulmonary, and adrenal involvement. The patient later passed away due to acute hypoxic respiratory failure. This case demonstrates rare sequelae of systemic AA amyloidosis of pulmonary hypertension and adrenal insufficiency.

## Introduction

Systemic amyloid A (AA) amyloidosis usually occurs in the setting of chronic inflammation when AA amyloid fibrils deposit in organs such as the spleen, liver, gut, and kidneys [1]. While adrenal glands are frequently found to have amyloid deposition, adrenal function is rarely affected [1]. Pulmonary amyloidosis is a rare manifestation of AA amyloidosis and may present asymptomatically as an incidental finding on imaging, but may also result in pulmonary hypertension [2-5]. We present a rare case of systemic AA amyloidosis resulting from chronic sialadenitis which manifested as pulmonary hypertension and adrenal insufficiency.

## Case presentation

**Figure 1:**
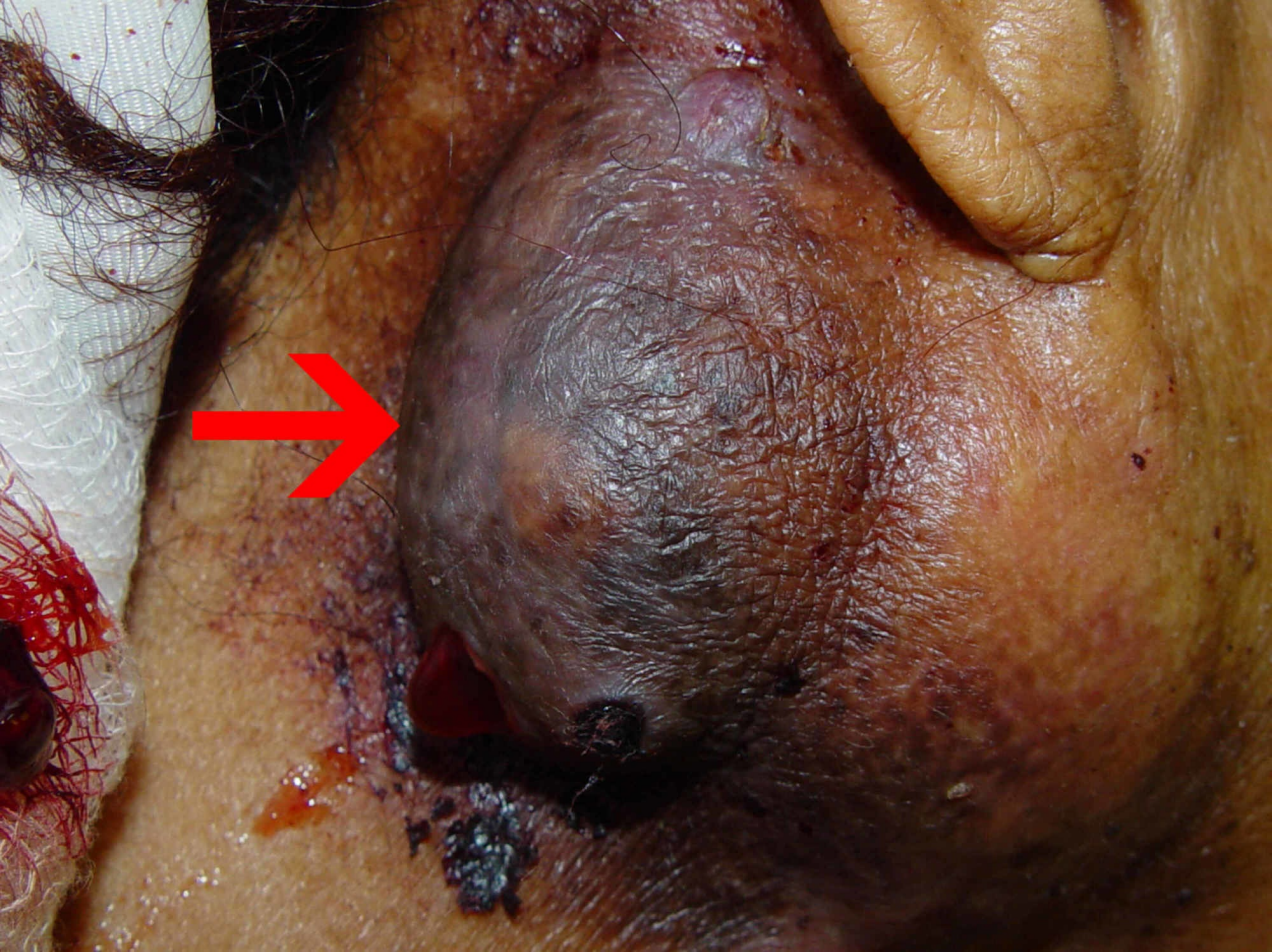
Right-sided neck mass of chronic sialadenitis (red arrow).

A 48-year-old African-American woman with a history of hypertension presented with a right-sided neck mass of 10 years with intermittent purulent discharge. She additionally reported three years of unintentional weight loss, fatigue, anorexia, constipation, night sweats, and chills. On presentation, her vital signs were within normal limits. On physical examination, the woman had severe bitemporal wasting, skin pallor, and a large right-sided neck mass with sanguineous discharge (Figure 1), but no lymphadenopathy. Cardiac and pulmonary examinations were normal; however, her abdomen was notable for hepatomegaly of approximately 18 cm and a palpable spleen. Initial laboratory investigations are presented in Table 1.

**Table 1:** Laboratory investigations on admission. MCV, mean corpuscular volume; AST, aspartate aminotransferase; ALT, alanine aminotransferase; ALP, alkaline phosphatase; GGT, gamma-glutamyl transferase; LDH, lactate dehydrogenase; INR, international normalized ratio; PTT, partial thromboplastin time

Test	Result	Reference range
Hemoglobin	5.8 d/dL	12.9-16.8 g/dL
MCV	71 fL	81.9-97.8 fL
Creatinine	2.7 mg/dL	0.6-1.4 mg/dL
Calcium	10.5 mg/dL	8.5-10.5 mg/dL
Glucose	60 mg/dL	65-110 mg/dL
Albumin	2 g/dL	3.8-5.2 g/dL
Total protein	8.5 g/dL	6.4-8.3 g/dL
Total bilirubin	1.6 mg/dL	0.2-1.2 mg/dL
AST	35 U/L	0-40 U/L
ALT	11 U/L	5-35 U/L
ALP	1200 U/L	20-120 U/L
GGT	84 U/L	3-60 U/L
LDH	238 U/L	85-210 U/L
Ferritin	80 ng/mL	23.90-336.20 ng/mL
INR	1.0	0.7-1.2
PTT	40 s	28-40 s

**Figure 2:**
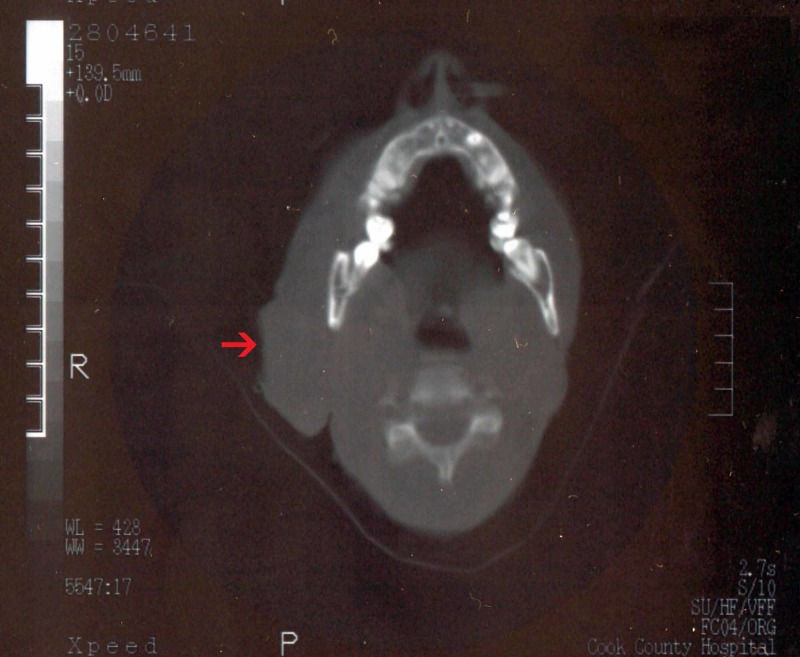
CT scan of the neck showing right-sided neck mass (red arrow).

CT scan of the neck showed a 4-cm cystic mass abutting the posterior aspect of the superficial lobe of the right parotid gland with multiple calcifications within the right parotid gland (Figure 2). Fine needle aspiration of the parotid mass revealed acinar cells of the salivary gland with focal atypia and severe inflammation suggestive of sialadenitis. While awaiting further workup, the patient left against medical advice.

Four days later, she was brought back to the hospital by her family after they found her confused. Upon this presentation, she was hypotensive, tachycardic and disoriented to place, time, and circumstances. CT scan of the head revealed no acute abnormalities. CT chest showed three right-sided pulmonary nodules and bilateral pleural effusions. Despite aggressive fluid resuscitation in the medical ICU, she remained hypotensive.

Investigations

Investigations were undertaken for hypotension. Serum chemistry was significant for a calcium level of 14.4 g/dL. Serum cortisol was 4.6 ug/dL (10-20 ug/dL) with no response to adrenocorticotropic hormone (ACTH) stimulation after 30 min (5.8 ug/dL) and one hour (6.9 ug/dL). Echocardiography showed a normal left ventricular ejection fraction and normal diastolic parameters. However, the estimated pulmonary artery systolic pressure was elevated at 50 mmHg with concomitant severe right ventricular dysfunction and right atrial enlargement.

**Figure 3:**
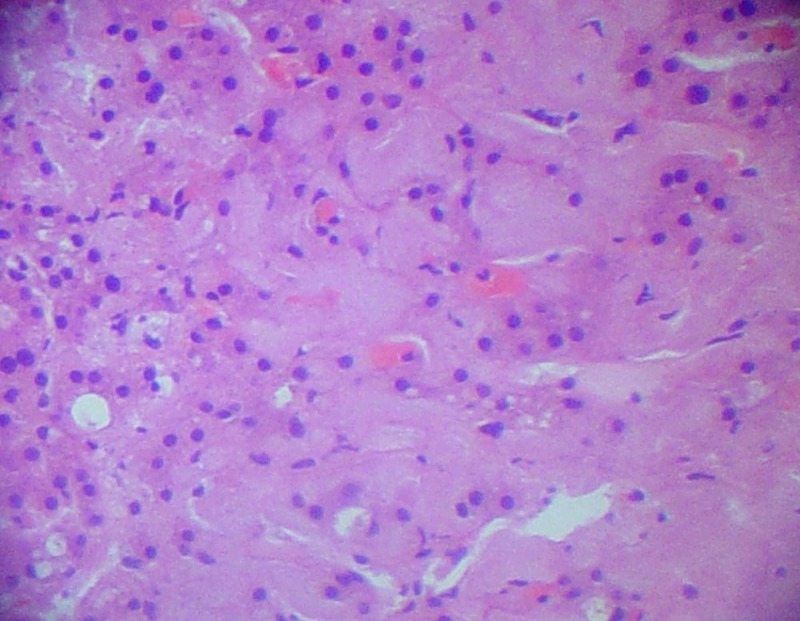
Hematoxylin and eosin stained liver biopsy specimen showing amyloid deposition.

Further workup showed normal serum and urine electrophoresis, negative HIV antibodies, anti-nuclear antibody, and anti-mitochondrial antibody. Parathyroid hormone level was normal at 58 pg/mL (ref: 10-65 pg/mL) and serum angiotensin converting enzyme (ACE) level was elevated at 141 U/L (ref: 8-52 U/L). Bone marrow biopsy showed hypercellular marrow, no atypical infiltrates or granuloma, and no abnormal clonal cells on flow cytometry. Core needle biopsy of the liver subsequently showed pale homogenous eosinophilic deposits in between hepatocellular and sinusoidal trabeculae, compatible with amyloid deposition (Figure 3).

Differential diagnosis

Lymphoproliferative disease such as lymphoma, nonlymphoproliferative metastatic cancer, sarcoidosis, disseminated tuberculosis, and disseminated fungal infection were amongst the diagnostic possibilities considered.

Treatment

The patient was started on stress dose intravenous hydrocortisone with subsequent improvement in blood pressure. She was subsequently transitioned to oral hydrocortisone and discharged in stable condition with outpatient follow-up.

Outcome and follow-up

Two months after discharge, the patient presented with five days of shortness of breath and cough and was found to be hypoxic. Chest X-ray showed increased burden of lung nodules in addition to multifocal lung consolidation. Intubation was offered to the patient in the setting of worsening respiratory distress and increasing supplemental oxygen requirements, however, she declined and passed away on the night of admission.

## Discussion

Amyloidosis is a systemic disease characterized by the deposition of mutated insoluble fibrils in extracellular tissue. Systemic AA amyloidosis occurs when the acute-phase reactant serum AA protein is misfolded and aggregated in the B-sheet configuration in the setting of prolonged inflammatory disease [1]. It is the most common cause of amyloidosis in the developing world, due to a high prevalence of chronic infections. It is less common than primary light-chain and wild-type transthyretin amyloidosis in Western countries where there is effective treatment for auto-immune disorders and a low incidence of chronic infections [1, 6]. While AA amyloidosis is known to occur in the setting of any chronic inflammatory disorder, its occurrence in the setting of chronic sialadenitis, as in the case described, is not well established. In a case series of 374 patients by Lachmann et al, the most common underlying disorder leading to AA amyloidosis was chronic inflammatory arthritis, followed by chronic sepsis and periodic fever syndromes [1].

The most common clinical manifestation of AA amyloidosis at the time of diagnosis is renal dysfunction (defined as > 500 mg protein excretion per day or serum creatinine concentration > 1.5 mg/dL) affecting approximately 97% of patients [1]. Hepatic deposition is common with up to 23% of patients having evidence of infiltration on serum amyloid P component (SAP) scintigraphy [7]. An alkaline phosphatase elevation of 1.5 times normal limits is considered diagnostic for hepatic involvement of amyloidosis [7], as was found in our patient. In the NEJM series, alkaline phosphatase (ALP) elevation was noted in 12% of patients, however, hepatic failure is not usually observed in AA amyloidosis [1, 8]. Splenic deposition is ubiquitous, however, rarely clinically apparent. Cardiac infiltration with subsequent cardiac failure may also occur, however, it only occurs sporadically in patients with AA amyloidosis [1, 6].

Pulmonary amyloidosis is a rare entity despite frequent deposition of amyloid in lung tissue on autopsy of patients with amyloidosis [9]. Most cases described have occurred in the setting of primary light chain (AL) amyloidosis, with isolated cases of wild type (TTR) and secondary (AA) amyloidosis [2-5, 9-10]. In a Mayo Clinic review of 55 patients with pulmonary amyloidosis, only two patients were diagnosed with secondary AA amyloidosis, one with familial Mediterranean fever (FMF) and another with bronchiectasis [2].

Pulmonary amyloidosis associated with AA amyloid may present incidentally on imaging with nodular lesions (“amyloidomas”) or a diffuse interstitial pattern [2-3]. Calatayud et al. described a 67-year-old woman with long-standing rheumatoid arthritis who was incidentally found to have a left-sided pulmonary nodule, which was followed with yearly CT. After multiple nodules were found two years later, one was resected and found to have pathological findings consistent with AA amyloidosis [4]. As in our case, patients with pulmonary AA amyloidosis may also concomitantly develop pulmonary hypertension. In one case, a 60-year-old Turkish woman presenting with cough was found to have diffuse interstitial infiltrates on CT and nephrotic syndrome. In search of an etiology, lung biopsy showed amyloid deposition in vascular walls and interalveolar septa. Immunohistochemical staining of amyloid from the lung biopsy, as well as a renal biopsy showed AA protein. An echocardiogram showed an elevated pulmonary artery pressure of 40 mmHg. She was eventually found to be homozygous for M69V mutation and diagnosed with FMF [3]. In a previous case, a 48-year-old woman with known FMF was evaluated for hypoxia with CT which found mediastinal adenopathy, a diffuse interstitial process with thickening of interalveolar septa, as well as many miniscule nodular changes. She was also found to have pulmonary hypertension, which was diagnosed via cardiac catheterization [10]. In a Mayo Clinic case series of patients diagnosed with pulmonary hypertension and amyloidosis, one of the five patients described also had FMF-related AA amyloidosis, as determined by liver biopsy. Pulmonary hypertension in amyloidosis is thought to result from amyloid deposition within vascular walls causing elevated pulmonary vascular resistance and endothelial dysfunction. It is a marker of advanced amyloidosis with a median survival time of 73 days in the Mayo series [5].

Adrenal glands are a frequent site of amyloid accumulation in AA amyloidosis, however, adrenal insufficiency is a rare outcome [1]. Adrenal uptake was evident in 154 (41%) of patients with AA amyloidosis studied in the 374 patients reported in NEJM, however, only five (3%) of those patients required treatment for adrenocorticoid insufficiency [1]. The incidence of acute adrenal crises in the setting of AA amyloidosis is unclear, however, two cases have been reported in FMF-related amyloidosis [11-12]. Clinicians should have a high index of suspicion for adrenal insufficiency in patients with secondary amyloidosis presenting with hypotension/shock.

## Conclusions

In conclusion, emphasis should be placed on treatment of chronic sialadenitis to avoid complications such as AA amyloidosis. Though rare, AA amyloidosis could result in adrenal failure and pulmonary hypertension. Patients with chronic sialadenitis and multiple organ involvement should be evaluated for amyloidosis to avoid downstream complications of the disease process. 
